# Identification and Management of Aggressive Meningiomas

**DOI:** 10.3389/fonc.2022.851758

**Published:** 2022-03-23

**Authors:** Bhuvic Patel, Rupen Desai, Sangami Pugazenthi, Omar H. Butt, Jiayi Huang, Albert H. Kim

**Affiliations:** ^1^ Department of Neurological Surgery, Washington University School of Medicine, St. Louis, MO, United States; ^2^ Department of Medicine, Division of Medical Oncology, Washington University School of Medicine, St. Louis, MO, United States; ^3^ The Brain Tumor Center, Siteman Cancer Center, Washington University School of Medicine, St. Louis, MO, United States; ^4^ Department of Radiation Oncology, Washington University School of Medicine, St. Louis, MO, United States

**Keywords:** meningioma, CNS tumors, chemotherapy, radiation therapy, immunotherapy, skull base surgery

## Abstract

Meningiomas are common primary central nervous system tumors derived from the meninges, with management most frequently entailing serial monitoring or a combination of surgery and/or radiation therapy. Although often considered benign lesions, meningiomas can not only be surgically inaccessible but also exhibit aggressive growth and recurrence. In such cases, adjuvant radiation and systemic therapy may be required for tumor control. In this review, we briefly describe the current WHO grading scale for meningioma and provide demonstrative cases of treatment-resistant meningiomas. We also summarize frequently observed molecular abnormalities and their correlation with intracranial location and recurrence rate. We then describe how genetic and epigenetic features might supplement or even replace histopathologic features for improved identification of aggressive lesions. Finally, we describe the role of surgery, radiotherapy, and ongoing systemic therapy as well as precision medicine clinical trials for the treatment of recurrent meningioma.

## Introduction

Meningiomas are the most common primary central nervous system (CNS) tumor, constituting more than 35% of adult brain tumors (1). At present, these tumors are classified by the World Health Organization (WHO) into three grades: WHO grade 1 (benign, representing the majority of all meningiomas), WHO grade 2 (atypical), and WHO grade 3 (malignant) (2). Although lower grade tumors are considered benign, these lesions can clinically behave aggressively. In a subset of individuals, low WHO grade meningiomas will recur despite multimodal management including surgical resection, radiation therapy, and systemic therapy (3). Studies with long follow-up have demonstrated recurrence rates as high as 47% after 25 years, but the role of WHO grade in recurrence is unclear, making it challenging to select patients who would benefit from adjuvant therapies (4). Indeed, emerging data suggest that many factors not previously included in the WHO grading schema can alter the prognosis of even benign WHO grade 1 lesions (5–10). As we will discuss, the recently released WHO 2021 classification marks a pivotal alteration in meningioma grading by incorporating for the first time key genomic alterations into the grading scheme (2, 11).

In this review, we highlight the incongruence between histologic grading and the clinical course of meningiomas, particularly aggressively behaving lesions. First, we describe illustrative case examples of meningiomas with different presenting WHO grade but uniformly aggressive clinical course. We then summarize more recently discovered histopathological and genomic features that may better predict meningioma aggressiveness. Finally, we describe the current surgical, radiotherapy, and targeted drug options available for treatment of aggressive, recurrent meningioma.

## Case Examples

While most meningiomas exhibit a benign clinical course and favorable response to treatment with surgery or radiotherapy, challenging cases are not uncommon. Few evidence-based treatment algorithms have been developed to address treatment-resistant meningiomas, in part due to the paucity of alternatives to the traditional treatments of surgical resection and radiotherapy (12). In this section we describe the clinical course and treatments used to treat three meningiomas that exemplify challenging lesions recalcitrant to treatment and that deviate from their expected clinical course based on the 2016 WHO classification scheme.

### WHO Grade 1 to WHO Grade 2

A 40-year-old woman presented with blurry vision in her left eye and was discovered to have a left frontal mass centered above the sphenoid wing that underwent Simpson Grade II resection and was diagnosed as a WHO grade 1 meningioma with low Ki-67 index and no brain invasion. Within a year, the patient’s meningioma recurred, with growth demonstrated on magnetic resonance imaging (MRI). During her second surgical resection, involved bone of the skull base near the orbital apex and roof was removed (Simpson Grade I). Despite the aggressive course, histopathological analysis once again demonstrated WHO grade 1 meningioma, with invasion of bone, and the patient underwent adjuvant fractionated radiotherapy to the resection bed.

The patient remained symptom-free for 14 years before experiencing worsening visual acuity, double vision with transient left eye deviation, and pain in her left orbit. MRI demonstrated a 2.5 cm diameter recurrence of her tumor, invading the left orbital apex and encasing the optic nerve. She experienced little improvement with a two-week course of prednisone and her symptoms progressed to left eye visual loss and proptosis over the course of a month before she underwent Gamma Knife stereotactic radiosurgery (SRS) as salvage therapy (15 Gy to the 50% isodose line).

The tumor initially decreased in size on serial MRI, but two years later the patient presented with epistaxis and sinonasal congestion, with tumor invasion of the sphenoid sinus, pterygopalatine fossa, and masticator space. She underwent tumor embolization followed by subtotal resection *via* expanded endoscopic endonasal approach, with histopathological analysis now consistent with WHO grade 2 meningioma with rhabdoid features and *NF2* mutation (genomic sequencing was not available at the time of prior resections). Within 3 months post-operatively, the size of the residual tumor increased, and by 6 months post-operatively the tumor filled the orbit and had increased from 1.8 cm to 4.5 cm in maximal diameter. Despite radical resection including a frontotemporal craniotomy, orbital exenteration and radial forearm free flap, the patient had multifocal tumor recurrence and over the course of 6 months underwent SRS twice and additional surgery for debulking and symptom relief. She was initiated on octreotide, pembrolizumab, and everolimus but was unable to tolerate the treatments due to skin rashes, thrush, and constipation. Ultimately, the patient elected to proceed with hospice care for her treatment-refractory meningioma and passed away soon thereafter.

### WHO Grade 2

A 54-year-old man presented with deteriorating right eye vision over the course of several years and trigeminal nerve distribution pain, and was found to have a large cavernous sinus, middle fossa, and infratemporal fossa mass. The patient underwent tumor embolization followed by craniotomy and subtotal resection, with a pathological diagnosis of WHO grade 2 meningioma with bony invasion, low MIB-1 index, no intratumoral necrosis, and no brain invasion. The residual tumor encasing the carotid artery was treated with fractionated radiotherapy (54 Gy in 30 fractions).

The patient was lost to follow up but presented 4 years later with progressive right-sided hearing loss, and his tumor was found to have invaded the right external auditory canal, middle cranial fossa, cavernous sinus, sphenoid sinus, and sella. The patient underwent a craniotomy for tumor resection with mastoidectomy and temporal bone resection, with residual tumor encasing the petrous carotid artery deemed too high-risk to resect. One year later, the residual tumor was found to have grown to involve the sphenoid sinus and left medial orbital wall. He underwent embolization and tumor debulking *via* a combined endonasal and transfacial approach. Eight months later, the patient presented with persistent epistaxis requiring embolization and was found to have extensive recurrence of his tumor for which he underwent endoscopic endonasal debulking once again. The pathological diagnosis after all his resections remained WHO grade 2 meningioma, with sequencing after his second surgery revealing only a *NF2* mutation.

During subsequent observation, the patient developed right eye blindness and left eye decline in visual acuity. He was deemed not to be a candidate for surgical or radiation therapies and therefore received therapy with compassionate use temsirolimus. Unfortunately, the tumor did not respond, and the patient developed side effects of severe hyperglycemia and eczematous dermatitis. Two years after his last surgery, the patient developed significant sinonasal disease and difficulty eating. A gastrostomy tube was placed for feeding and the patient underwent palliative debulking of tumor in his sinonasal cavity before transitioning to hospice care and expiring 3 months later.

### WHO Grade 2 to WHO Grade 3

A 49-year-old female was diagnosed with a left spheno-orbital meningioma after presenting with left eye proptosis and underwent tumor embolization followed by craniotomy with gross total resection (Simpson Grade I) and adjuvant fractionated radiotherapy with a dose of 54 Gy. Her histopathological diagnosis at that time was WHO grade 2 meningioma with increased mitotic activity of 8 mitoses per 10 high powered fields and bony invasion. After 10 years of follow-up with serial MRI, she was found to have a thin area of recurrent tumor, which was treated with SRS (20 Gy to the 50% isodose line).

Three years later, the patient’s tumor was found to have slightly increased in size on annual MRI scan. This was initially managed with continued close serial observation, but one year later the patient presented with proptosis, inferior displacement of the left globe and diminished left eyelid function with increase in tumor size. Subtotal surgical resection was performed, and histopathological diagnosis remained WHO grade 2 meningioma, this time with Ki-67 index of 24.8%, and *SMO* mutation detected on genomic sequencing. Her tumor increased in size two years later, and she was initiated on octreotide and everolimus. She tolerated octreotide well but developed mucositis, elevated liver enzymes, anemia, and hyperlipidemia necessitating decreased dosing of everolimus.

This therapy was continued for 2 years, until serial MRI showed tumor progression and the patient’s left eye visual acuity began to decline. She was enrolled in an institutional clinical trial of proton beam therapy [20 Gy relative biological equivalents (GyRBE) in 5 fractions], neoadjuvant avelumab (6 doses), and surgical resection, including complete orbital exenteration, near total tumor resection, and left thigh free flap for skull base reconstruction. Histopathological examination of the tissue was now consistent with WHO grade 3 meningioma with foci of rhabdoid and papillary arrangements, necrosis, brain invasion, and 23 mitoses per high powered field. The patient has been followed with serial MRI showing stable residual disease one year postoperatively.

## Histopathology & Genetics

Although most meningiomas are easily diagnosed with computed tomography or magnetic resonance imaging, histopathological analysis of tumor tissue remains the cornerstone of tumor subtyping and grading. In recognition of the value of molecular features in brain tumor subtyping, the 2016 World Health Organization Classification of Tumors of the Central Nervous System for the first time integrated molecular parameters in addition to histological features for classification of many CNS tumors (13). Unfortunately, no molecular features were included for an integrated diagnosis of meningiomas, and, aside from including brain invasion as a histological criterion for WHO grade 2 meningiomas, no changes were made to meningioma grading (13). However, certain histopathological features, such as necrosis, have been found to predict more aggressive treatment-resistant behavior, and there is a growing body of evidence that specific molecular features may more clearly delineate meningioma subtypes that better correlate with clinical course (5–7, 14–16). The 2021 World Health Organization Classification of Tumors of the Central Nervous System integrated these newer data in its recent revision of meningioma taxonomy, with the addition of molecular markers denoting a higher grade even in the absence of traditional anaplastic features on histology (2, 11).

### Histopathology

The first internationally agreed upon subtypes of meningioma were characterized by Bailey and Cushing based on histopathological features (17). Histopathological analysis continues to be the basis for characterization of meningiomas, with the current WHO grading system still retaining 15 meningioma subtypes. While previous WHO revisions further subclassified 9 subtypes as WHO grade 1, 3 as WHO grade 2, and 3 as WHO grade 3, tumor grade is no longer coupled to subtype in the 2021 revision (2, 11).

Each update to the WHO classification has resulted in dramatic shifts in the proportion of meningiomas of each grade and have improved upon the correlation between WHO grade and clinical course. For example, the 2000 and 2007 updates to the WHO classification resulted in the number of meningiomas graded as WHO grade 2 increasing from 5% to 20% (18). With the addition of brain invasion as a criterion for WHO grade 2 meningiomas in the 2016 update, this proportion is approximately 35%, and has increased further with the WHO 2021 classification upgrading chordoid and clear cell meningioma from grade 1 to grade 2 (1, 2).

More recent studies focusing on WHO grade 2 meningiomas have uncovered histopathological features that identify a more aggressive clinical subtype within this group of tumors, further complicating treatment decision making for patients diagnosed with grade 2 tumors. In 2014, Sun et al. reported that tumor necrosis predicted radiation resistance in WHO grade 2 meningiomas that were sub-totally resected (15). Additionally, the co-occurrence of brain invasion and high mitotic index and the co-occurrence of brain invasion and necrosis have both been reported to increase the risk of radiotherapy resistance and recurrence in WHO grade 2 meningiomas (16, 19). Ultimately, despite these improvements in correlating histopathological features with clinical outcomes, such features remain subject to high interobserver and sampling bias, increasing the need for more reliable molecular markers that predict tumor behavior and therapy resistance.

### Genomic Analysis

The meningioma genomic landscape has been an area of significant investigation as early as 1994 when Ruttledge et al. first highlighted the prevalence of mutations in the *NF2* gene, located on chromosome 22q (20). At the time, it was well known that loss of heterozygosity (LOH) on chromosome 22 was present in up to 80% of meningiomas. Sequencing of the *NF2* tumor suppressor gene in tumors with chromosome 22 LOH revealed that a significant number of tumors harbor inactivating mutations of *NF2* (20). Mutations in *NF2* in the absence of chromosome 22 LOH are rarely observed in meningiomas, corroborating the two-hit hypothesis for the function of the *NF2* gene as a tumor suppressor in spontaneous meningiomas.

**Figure 1:**
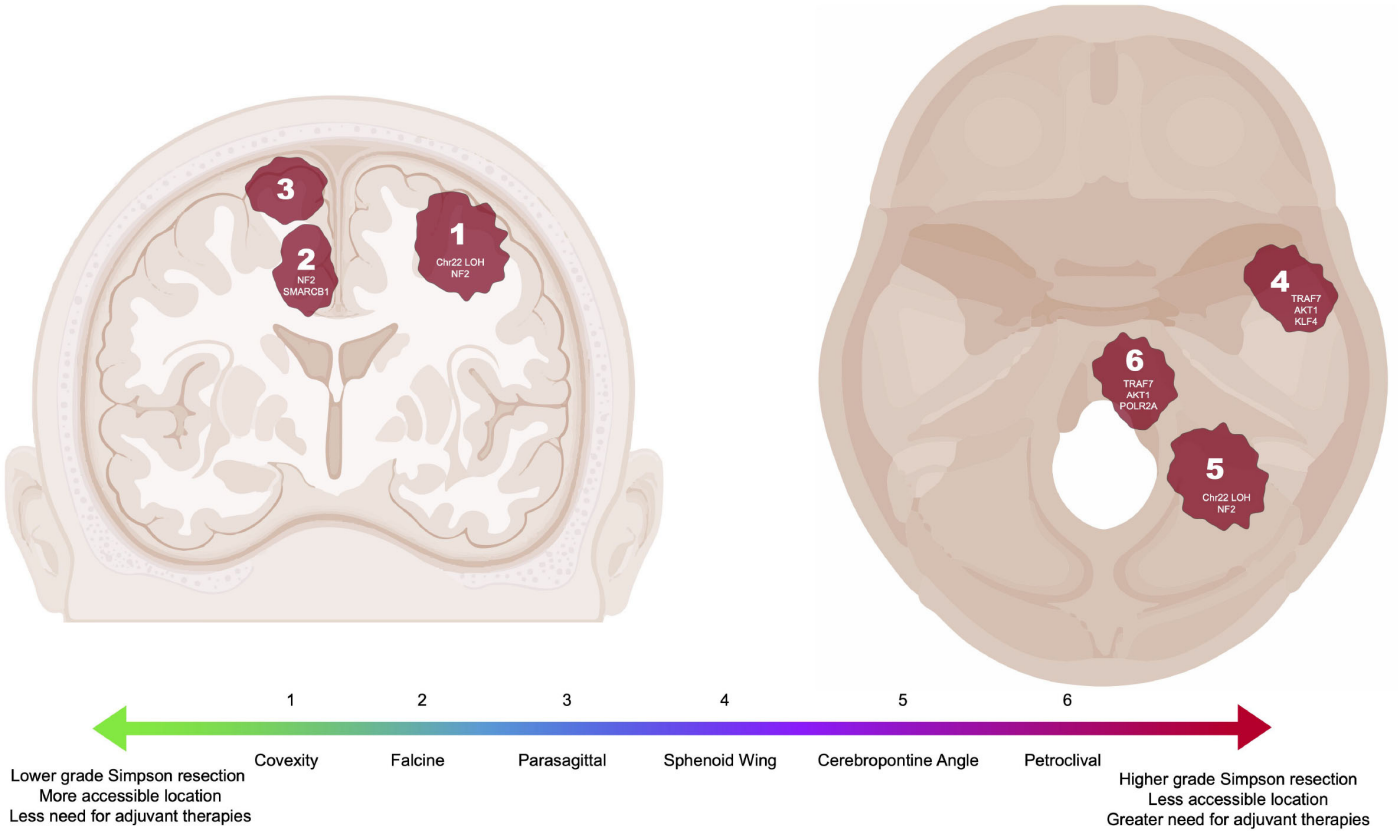
Common intracranial locations of meningiomas highlighted in this review with associated DNA driver mutations or chromosomal loss (6, 14, 23, 24). Locations correlated to a generalized scale ranging from less (green) to more (red) complicated to resect and manage. Meningioma locations not pictured include clinoid, foramen magnum, cavernous sinus, suprasellar, and tentorial.

Notably, higher grade tumors more often harbor *NF2* mutations in addition to large-scale chromosomal abnormalities and overall higher mutational burden (14, 21, 22). Further investigation of *NF2* mutations in meningiomas has also revealed that these mutations are generally associated with convexity meningiomas rather than meningiomas of the anterior skull base (**Figure 1**) (14, 23). Merlin, the protein encoded by *NF2*, is as an intracellular scaffolding protein that indirectly links F-actin, transmembrane receptors and intracellular effectors. It has been shown to function as a tumor suppressor by inhibiting cell growth through contact inhibition and resultant activation of the Rac1 pathway in the setting of high cell density. Loss of merlin function therefore results in loss of contact inhibition of growth. *NF2* mutation also results in activation of the Hippo, Notch, phosphoinositide 3-kinase (PI3K)/AKT, mammalian target of rapamycin (mTOR), and Ras/mitogen-activated protein kinase (MAPK) pathways with resultant increase in cell proliferation (25–29). As described later, these insights into the molecular biology of meningiomas have identified targets for pharmacological agents such as tyrosine kinase inhibitors that subsequently also decrease activation of the PI3K, mTOR, and ERK pathways.

Given that 40% of meningiomas do not have mutations in *NF2*, more recent investigation has focused on identifying other drivers of meningioma tumorigenesis using next-generation sequencing techniques that facilitate genome-wide sequencing in large cohorts of patients. In 2013, two seminal studies evaluating key genetic characteristics of meningioma were published (14, 30). Clark et al. identified mutations in *TRAF7* (*tumor necrosis factor [TNF] receptor–associated factor 7*), *KLF4* (*Kruppel-like factor 4*), *AKT1* (*v-akt murine thymoma viral oncogene homolog 1*), and *SMO* (*smoothened*) after sequencing a cohort of 300 WHO grade 1 and 2 meningiomas (14). Interestingly, these additional mutations identified three non-overlapping groups of tumors with distinct locations: those with chromosome 22 LOH and *NF2* mutations occurring along the convexities and posterior skull base, those with mutations in *SMO* occurring in the midline anterior skull base, and those with mutations in *TRAF7*, *AKT1*, and/or *KLF4* occurring in the sphenoid wing and floor of the middle fossa (**Figure 1**) (14). Identification of these non-*NF2* driver mutations revealed that the meningioma genomic landscape was more diverse than previously assumed, leading to the identification of additional meningioma driver mutations including *POLR2A*, *SMARCB1 germline variants (including SMARCE1)*, *AKT3*, *PIK3CA*, *PIK3R1*, *PRKAR1A*, *SUFU*, and *BAP1* (21, 24, 30–33). At the same time, Brastianos et al. performed genomic sequencing of 17 meningiomas with further targeted sequencing on an additional 48 meningiomas, finding WHO grade 1 meningiomas have significantly lower rates of genomic disruptions than either systemic tumors or WHO grade 2 or 3 meningiomas (30). In the discovery set, some Grade 1 meningiomas were found to have copy number loss on chromosomes 1p, 7p, 14p, and 19 with copy number gains on chromosomes 5 and 10, while higher grade tumors were associated with copy number loss of chromosomes 10q and 14q. Targeted gene analysis identified non-synonymous mutations in *NF2* (the most common alteration), *KDM5C, SMO, AKT1, RGPD3*, and *CD300C*. The specific *SMO* mutations were previously known oncogenic mutations in basal cell carcinoma and desmoplastic medulloblastoma and were only found in meningiomas without *NF2* alterations. Similarly, the *AKT1* mutations were oncogenic mutations previously described in breast, colorectal, and lung cancer and were mutually exclusive with *NF2* or *SMO* mutations in meningioma. In validation cohorts, the *AKT1* and *SMO* mutations were observed in skull base and higher grade meningiomas. Together, the findings from Clark et al. and Brastianos et al. laid the foundation for the inclusion of genomic alterations in the 2021 WHO classification.

The genomic landscape specifically of WHO grade 3 meningiomas has historically been less well characterized. To address this question, Bi et al. analyzed 134 high-grade meningiomas. In their cohort of high grade meningiomas, most tumors were characterized by *NF2* mutations, with very few tumors having mutations in *TRAF7, KLF4*, *AKT1* and *SMO*, suggesting that high grade meningiomas have few targetable genetic mutations. Associations were also reported between *AKTI/PIK3CA* mutations and meningothelial subtype, *NF2* mutations and fibroblastic subtype, and *TRAF7/KLF4* mutations and secretory subtype. Bi et al. also found that high grade lesions were characterized by increased copy number alterations, and, interestingly, low grade lesions that progressed to high grade meningiomas exhibited patterns of genomic disruption similar to high grade meningiomas and have been associated with activating *TERT* promoter mutations (21, 34). Presence of a *TERT* promoter mutation is further associated with progression and poor survival (35, 36). This observation combined with other groups demonstrating a strong association between homozygous deletion of *CDKN2A/B* or *BAP1* mutations and aggressive clinical outcome led to a significant revision in the 2021 WHO criteria (2, 37–39).

While the 2021 classification recommends considering sequencing, it is not required for diagnosis (2). Nonetheless, the current criteria now integrates driver mutations such as *NF2*, *AKT1*, *SMO*, and *PIK3CA* for conventional, *TRAF7* and *KLF4* for secretory, *SMARCE1* for clear cell, and *BAP1* for rhabdoid subtypes (2, 11). Furthermore, a meningioma harboring either a *TERT* promotor mutation or homozygous deletion of *CDKN2A/B* is classified as a grade 3 anaplastic tumor, regardless of histologic grade (11).

### Expression Profile and Epigenomic Analysis

Given that WHO grade and DNA mutations do not optimally predict the clinical behavior of meningiomas, recent studies have used several molecular analysis techniques to create classification schemes more aligned with meningioma clinical course (**Table 1**). Many groups have hypothesized that chromatin structure and gene expression profiles, governed largely by DNA methylation, might be more useful in this regard (40–42). For example, the loss of H3K27 trimethyl (H3K27me3) identified by immunohistochemistry has been corroborated as a marker of poor survival and shorter time to recurrence, specifically in Grade 2 meningioma (43, 44).

**Table 1:** A comparative representation of studies describing molecular reclassification of meningiomas.

	Bayley et al, 2022 (10)			Nassiri et al, 2021 (7)				Maas et al, 2021 (8)		
	MenG A	MenG B	MenG C	Group 1	Group 2	Group 3	Group 4	Subtype 1 (Low)	Subtype 2 (Int)	Subtype 3 (High)
**TRAF7**										
**AKT1**										
**KLF4**										
**SMO**		N/A						N/A	N/A	N/A
**POLR2A**								N/A	N/A	N/A
**SMARCB1**								N/A	N/A	N/A
**Chr22q Loss**										
**NF2**										
**Chr1p Loss**										
**TERT**	N/A	N/A	N/A							

Within each classification scheme, meningioma subgroups are ordered from left to right based on increasingly worse progression free survival. Genetic mutations and chromosomal losses were compared across each subgroup with black shading indicating predominant mutation/loss in that group, empty cell indicating that the mutation/loss was tested for but was not present or significantly less predominant in that group, and N/A indicating that the mutation/loss was not tested for or not reported in that study.

In 2017 Sahm et al. performed genome-wide methylation analysis of 497 meningiomas across all WHO grades and 309 extra-axial tumors that mimic meningiomas histologically (5). Unsupervised clustering not only segregated meningiomas from the other tumors but also identified six clinically relevant methylation classes of meningiomas. Three classes clustered together and were defined as methylation class benign 1 to 3 (MC ben-1 to MC ben-3) based on having a more benign clinical course. Two classes, defined as methylation class intermediate A and B (MC int-A and MC int-B) had an intermediate progression-free survival, and the final class, methylation class malignant (MC mal), had a markedly poor progression-free survival. Notably, while WHO grade 1 meningiomas were enriched in the benign methylation classes and WHO grade 3 meningiomas in the malignant methylation class, WHO grade 2 lesions, which often have a heterogenous clinical course, were scattered across all but one of the methylation classes. Furthermore, *NF2* mutations were found in at least 30% of all methylation classes except for MC ben-2. Consistent with findings from genomic sequencing studies, non-*NF2* and *NF2* mutations occurred almost mutually exclusively, with non-*NF2* mutations being enriched in MC ben-2, while *NF2* mutations were rare in this group (5). Taken together, these findings suggest that methylation classes are superior to WHO grade for predicting clinical behavior, especially in the case of WHO grade 2 tumors, and that although *NF2* mutational status may not be an entirely specific predictor for clinical behavior, non-*NF2* driver mutations may be useful in identifying meningiomas with a more benign clinical course.

In 2019 Patel et al. reported their classification scheme based on combined bulk RNA sequencing and whole exome sequencing analysis of 160 meningiomas (6). They too reported 3 molecular subgroups, named type A, B, and C that predicted recurrence more reliably than the WHO grading schema. Interestingly, they found that more than half of the tumors in their most aggressive subgroup (type C) were predicted to be benign by WHO grading criteria. Like prior studies, the least aggressive tumors (type A) were found to have no notable copy number alterations while the most aggressive tumors (type C) were found to have the greatest rates of chromosome 22q and 1p losses. Importantly, gene set enrichment analysis of type B and type C tumors revealed loss of PRC2 complex function in type B and loss of DREAM complex function in type C tumors, insights which might guide targeted treatment strategies in the future (6).

More recently, Nassiri et al. performed an integrative analysis of 121 meningiomas with methylation array, bulk RNA sequencing, and whole exome sequencing analysis to develop an integrated classification system to better predict outcome than the WHO grading system (7). They identified 4 molecular subgroups of meningioma, MG1-4, and designated each subgroup based on pathway analysis of enriched genes: immunogenic (MG1), benign *NF2* wild-type (MG2), hypermetabolic (MG3), and proliferative (MG4). Interestingly, mapping drugs to target-enriched genes identified possible drug candidates for specific meningioma subtypes. For example, the histone deacetylase inhibitor vorinostat, which mapped to the MG4 subtype, was found to specifically decrease the viability of the MG4 meningioma cell line *in vitro* and decrease the size of MG4 xenografts *in vivo*, highlighting the value of molecular analysis of meningiomas both in classification and in development of novel therapies (7).

Maas et al. and Bayley et al. have similarly created meningioma classification systems that integrate a combination of methylation array data, copy number alterations, DNA mutations, and histopathological findings to better stratify patients (**Table 2**) (8, 10). Importantly, Maas et al. demonstrated that copy number alteration data can readily be inferred from methylation arrays, thus streamlining the molecular diagnostic workup of meningiomas, although they also provide alternative assays (targeted gene analysis or FISH) for stratification depending on resource availability (8). Bayley et al. combined DNA methylation, RNA-seq, and cytogenic profiling on WHO grade 1 and 2 meningiomas yielding three subgroups of meningiomas, two malignant and one benign. Notably chromosome 1p loss was strongly correlated with malignant tumors (10). These integrated models yielded a greater accuracy for prognosis compared to RNA-sequencing and cytogenic profiling or DNA methylation alone.

**Table 2:** Comparison of contemporary aggressive meningioma prognostication.

	Driver Classification (9)	Maas Classification (8)	RTOG 0539 (45)	WHO 2021 (2)
**CNV**				
1p				
3p				
4p/q				
6p/q				
10p/q				
14q				
18p/q				
19p/q				
CDKN2A/B				
**Mitoses**				
4 to 19				
>20				
**EOR**				
GTR				
STR				
**Tumor Volume**				
0-25 cc				
>25cc				
**Recurrence**				
rimary				
Recurrent				
**WHO Grade**				
1				
2				
3				
**Methylation Profile**				
Ben				
Int				
Mal				
**Genetic Alterations**				
SMARCE1				
BAP1				
KLF/TRAF7				
TERT				
**Histology**				

Key factors of four contemporary meningioma grading schemata, including genetic and epigenetic alterations, histologic characteristics, and clinical characteristics, are compared. Within each classification scheme, black shading indicates use of the factor in the prognostication score.

Meanwhile, Driver at al. incorporated 15 targeted, high-risk molecular alterations (13 chromosomal alterations and loss of *CDKN2A/B*) with histologic (presence of frequent mitoses) and clinical (extent of resection, tumor volume, and recurrence status) factors to stratify meningioma. This classification system highlights the importance of incorporating *CDKN2A* mutations, with their classification system resulting in reclassification of 32% of tumors into either a higher or lower risk integrated grade compared to their WHO grade (9). Taken together, these recent studies suggest that a combination of molecular and histopathological properties need to be considered for accurate stratification of meningiomas. Indeed, use of machine learning techniques may allow for inclusion of even more information, such as MRI characteristics of tumors, into intergrated grading schema that more accurately stratify meningiomas (46).

## Clinical Management

Active meningioma management includes surgical resection, radiation therapy, and pharmacological options (47, 48). Observation is another option, generally reserved for small, asymptomatic, or incidental lesions and for patients that are deemed poor candidates for other therapeutic options (49). These patients are typically monitored with serial MRI scans. Tumor growth or symptom progression can indicate that observation has failed, and additional treatment may be necessary. In a retrospective study of 244 patients, Oya et al. demonstrated tumor diameter at diagnosis greater than 25 mm, MRI T2 signal hyperintensity, absence of calcification, and edema predicted tumor growth (50). Additional retrospective studies validated these findings and demonstrated tumors > 40 mm at diagnosis and with initial volumetric growth rates of 20% per year are highly likely to progress (51–53). Presence of focal or diffuse calcification is perhaps one of the strongest preoperative radiographic predictors that a meningioma is unlikely to recur, demonstrating 0% recurrence rate in one retrospective study of 101 patients, compared to nearly 21% recurrence rates in meningiomas without calcification observed (54). While many patients with meningioma under observation are asymptomatic, temporarily mitigating mild symptoms is possible with low-dose steroids to alleviate edema and antiepileptic medications for patients that present with seizure.

### Surgery

With symptomatic lesions, tumor progression, or mitigating factors such as patient preference, an active management strategy is often required. For patients without significant medical comorbidities, surgical resection is considered first-line treatment and can often be curative. Selecting a surgical approach is a nuanced decision based on the specific meningioma location that must balance surgical risk with a need to achieve complete resection as described by the Simpson Grading Scale, defined by removal of the tumor with tumor-infiltrated dura, bone, and venous sinuses (47, 55, 56). Fundamentals of meningioma surgery are based on the general principle that they are extra-axial lesions, and bone must be removed to permit sufficient exposure of the lesion and minimize injury to surrounding neurovascular structures. The meningioma is first devascularized at its base to minimize bleeding, the core is debulked, and the now malleable capsule dissected from neurovascular structures. In select cases, preoperative embolization may aid devascularization. Tumor removal can be significantly more complicated for meningiomas with close involvement of cranial nerves or a venous sinus. Gross total resection of a meningioma is highly dependent on the consistency of the tumor, its involvement with surrounding structures, and tumor shape (57–59).

The location of a meningioma greatly affects a surgeon’s ability to achieve complete resection, with increasing complexity, for example, for convexity, parasagittal, sphenoid wing, cerebellopontine, and petroclival meningiomas (**Figure 1**). Convexity meningiomas typically present to the surface of the brain, placing fewer neurovascular structures at risk during dissection. Thus, given a generally low surgical risk, complete resection represents the standard of care for both initial surgical resection and for recurrence of aggressive convexity meningiomas. Similarly, falcine meningiomas that by definition arise from the falx but do not involve the superior sagittal sinus can generally be completely resected, frequently by an interhemispheric approach (60). In contrast, parasagittal meningiomas can abut or even invade the superior sagittal sinus, limiting a surgeon’s ability to achieve a gross total resection without incurring the risks associated with sinus thrombosis and venous infarction. Such cases require close monitoring of residual tumor for progression, with consideration for adjuvant therapy for clinically aggressive lesions (61, 62).

Clinoidal, sphenoid wing, and spheno-orbital meningiomas can also be more technically challenging, particularly with increasing meningioma size and more medial location along the sphenoid wing, given proximity to the optic nerve, internal carotid artery and its branches, and the cavernous sinus (47, 63). Such tumors may be amenable to frontotemporal craniotomy, an eyebrow approach, or in some cases a TONES (TransOrbital NeuroEndoscopic Surgery) approach to remove the lesion. Additional bony removal to improve resection of larger, more invasive lesions might include anterior clinoidectomy, optic nerve decompression, orbitozygomatic osteotomy, and removal of hyperostotic, tumor-infiltrated bone. Complete resection of aggressive, recurrent lesions may be limited by encasement of critical structures such as the internal carotid artery or infiltration into the cavernous sinus. Fortunately, adjuvant treatment of such lesions can yield control rates as high as 70%, although more aggressive approaches such as carotid bypass or cavernous sinus entry to remove additional tumor should be considered in certain cases (64).

Meningiomas localized within the cerebellopontine angle are associated with further operative risk due to proximity to the brainstem, cranial nerves, and high-flow vasculature. The operative corridor to the cerebellopontine angle depends on size of the meningioma and relationship to the cranial nerves. Possible approaches include the retrosigmoid craniotomy and posterior petrosal approach, among others. A case study of 34 patients with cerebellopontine angle meningiomas demonstrated a 35.3% rate of cranial nerve deficits postoperatively, particularly when tumor is greater than 3 cm or extends into the jugular foramen (65). Given the critical structures surrounding the cerebellopontine angle, complete resection might not be possible, particularly with significant extension into the jugular foramen or with brainstem invasion. Tentorial meningiomas, which represent 3-6% of intracranial meningiomas, represent a similarly technically complex subset of meningiomas. Depending on the location, surgical approaches include the subtemporal, suboccipital, or supracerebellar infratentorial approaches (66).

Petroclival meningiomas, originating at the upper clival dura medial to the trigeminal nerve, represent one of the most technically challenging meningiomas to treat surgically, given their ventral location in relation to the brainstem and involvement of multiple cranial nerves, with high rates of surgical morbidity (67). Variants such as the sphenopetroclival meningiomas can further extend into the middle fossa and invade the cavernous sinus. Complete resection of these tumors is often not possible and a combination of approaches, such as retrosigmoid, presigmoid, subtemporal transtentorial transpetrosal, and pretemporal transcavernous approaches, may be needed. Recent advances in endoscopy have opened a new avenue for resection, with the endonasal corridor providing direct access to the ventrally located meningioma *via* the clivus, reducing retraction on the brainstem or cranial nerves and greatly improving extent of resection (68–71).

Similarly, meningiomas of the anterior midline skull base (e.g. parasellar or olfactory groove) may be accessed by either expanded endoscopic approach or open craniotomy, often through frontotemporal or subfrontal approaches (72, 73). The expanded endoscopic approach is an increasingly viable option providing early visibility of the anterior fossa with minimal brain retraction and provides an opportunity for early decompression of the optic canal to reduce the risk of optic nerve injury. While such approaches are associated with increased risk of anosmia and cerebrospinal fluid (CSF) leak, advent of the vascularized nasoseptal flap has vastly reduced the rate of CSF leak.

Other locations of intracranial meningiomas are described in the literature but are less commonly observed and are therefore not comprehensively discussed in this review. For example, intraventricular meningiomas, which represent less than 3% of intracranial meningiomas, account for up to 15% of adult intraventricular neoplasms and can present with either mass effect or obstructive hydrocephalus; the most common left trigonal location is often approached through a superior parietal lobule or occipital avenues (74). Surgical approach for each subset of intracranial meningioma not discussed must be tailored to maximize resection while minimizing risk of injury to vasculature, cranial nerves, and brain parenchyma.

### Radiation Therapy

In elderly or medically ill patients, for surgically inaccessible tumors, or as an adjunct to open surgery, stereotactic radiosurgery is a treatment option that is particularly effective for smaller lesions, with 5 year progression-free survival rates ranging from 86-100% (18). Although surgery is widely considered first-line treatment for symptomatic or progressive meningiomas in a healthy patient population, complex tumors that are closely involved with critical neurovascular structures may not permit complete resection. Particularly for aggressive meningiomas, residual tumor, such as that left within the superior sagittal sinus, cavernous sinus, or encasing cranial nerves, is associated with a 5-year recurrence rate greater than 60% (75). Therefore, adjuvant therapy must be considered for these lesions and is considered standard of care even after complete resection for aggressive or grade 3 lesions (76).

Small (< 3 cm) WHO grade 1 meningioma may be treated with single- or multi-session radiosurgery, though this is not commonly used in higher-grade meningiomas except in cases of repeat irradiation (77–79). For larger meningiomas of high grade or with aggressive features, fractionated radiotherapy alone or in addition to surgery is often recommended, typically 54 Gy for grade 1 and 59.4 – 60 Gy for grade 2-3 lesions. Fractionated radiosurgery following subtotal resection in patients with WHO grade 1 meningiomas demonstrated 5-year progression free survival (PFS) of 91%, compared to 52% of patients that had subtotal resection alone (80). Similar comparisons in WHO grade 2 and 3 patients demonstrated significant increase in median PFS from 37 months to 64 months with addition of adjuvant fractionated radiotherapy, although this benefit is likely reduced in aggressive and recurrent meningiomas, which may be identified based on the presence of intratumoral necrosis or brain invasion, as previously described (81–83).

The efficacy of radiation therapy is highly relevant for surgically complex tumors where gross total resection is not possible. Large retrospective analyses of symptomatic patients with petroclival, cavernous sinus, and cerebellopontine meningiomas reveal tumor control by radiation therapy in greater than 90% of cases with significant improvement in cranial nerve function (46.5%), particularly in petroclival and cavernous sinus meningiomas (84, 85). These findings were replicated in a series of retrospective analyses meta-analyses specifically looking at patients with cavernous sinus meningiomas treated with fractionated radiotherapy, demonstrating a local control rate of approximately 90% at 10 years with cranial nerve deficit improvement in 26-45% of patients and a 10% rate of new cranial nerve dysfunction (86–90). Clinical trials further investigating the application of fractionated radiotherapy in the treatment of meningioma are ongoing with encouraging results. For example, preliminary results from RTOG 0539, a phase II clinical trial in which patients were assigned radiation treatment protocols based on grouping into low, intermediate and high-risk groups by extent of resection, WHO grade, and recurrence status indicate a PFS of 94% for intermediate- and 59% for high-risk meningiomas treated with adjuvant fractionated radiotherapy (45, 91). A lingering question is the need for adjuvant radiation therapy following gross total resection of an intermediate grade meningioma, and may be answered by an ongoing phase 3 clinical trial (NCT03180268). Of note, inclusion in this trial requires pathologic diagnosis of WHO grade 2 meningioma according to the 2016 criteria, and therefore may not entirely assess the genetic and epigenetic subclassifications described in recent years and incorporated into the WHO 2021 classification.

### Systemic Therapy

Medical management for meningioma is typically reserved as salvage therapy in aggressive, recurrent cases without surgical or radiotherapeutic options. Unfortunately, there is a lack of large-scale positive controlled trials on which to base recommendations, highlighting the importance of ongoing clinical trials. Instead, recommendations are based on small-scale studies evaluating a wide variety of drug classes. These include recombinant antibodies (such as the anti-angiogenesis drug class), small peptides (e.g. somatostatin analogues), and a range of small molecule targeted therapies (48, 92–94). Traditional cytotoxic agents have had limited success (95, 96).

The NCCN guidelines (version 2.2021) for the treatment of recurrent meningioma has four category 2 recommended treatments: bevacizumab (2A), sunitinib (2B), a combination of bevacizumab with everolimus (2B), and somatostatin analogue (2B, “useful in certain circumstances”). Here we first review the NCCN recommendations for different salvage therapies before discussing additional treatment options.

Therapies targeting angiogenesis predominately affect the Vascular Endothelial Growth Factor (VEGF) pathway. A ten-fold elevation of VEGF levels have been reported in high grade as compared to low grade meningiomas (97). Vasogenic edema associated with meningiomas likewise is correlated with tumor VEGF expression levels, suggesting promise for therapies targeting angiogenesis in certain meningiomas (98). Drugs directly and indirectly targeting this pathway include bevacizumab (targeted inhibition of VEGF-A), vatalanib (VEGF/PDGF receptor inhibition), and sunitinib (non-specific tyrosine kinase inhibitor) (48, 95). Of these agents, bevacizumab is the best studied, with reported median PFS ranging from 6 to 15 months across several retrospective and prospective phase 2 studies (94, 99–101). As summarized by Graillon et al. in their recent review, the majority of these studies are small, enrolling between 8 and 38 patients with grade 2 or 3 meningiomas (94, 100). This combined with heterogeneity of study populations between studies warrants caution when interpreting consensus guidelines. When compared to a range of systemic agents as part of a retrospective study, Furtner et al. also noted those receiving bevacizumab demonstrated an 80% reduction in tumor diameter and 107% reduction in peritumoral edema (95). While promising, a prospective study by Furuse et al. suggests this may be due to bevacizumab treating post-radiation intraparenchymal radiation necrosis rather than targeting viable tumor (100). Bevacizumab is the only NCCN 2A recommended systemic treatment for recurrent meningioma. Likewise, the European Association of Neuro-Oncology (EANO) recommends bevacizumab in cases without alternative local treatment options, with a European evidence level of III (48, 102).

One prospective phase 2 and one retrospective study have examined sunitinib in recurrent meningiomas, enrolling 36 and 11 patients respectively (103, 104). Kaley et al. reported expression of VEGF-R2 in high grade meningiomas was associated with a median PFS of 1.4 months compared to 6.4 in patients who lacked its expression. Unfortunately, hemorrhages were observed in 4 of 36 patients on the study (two grade 3, one grade 4, and one grade 5), with additional thrombotic microangiopathy noted in 2 patients (103). More recently, Cardona et al. reported a median PFS of 9.1 months in eleven patients treated with sunitinib, notably without reports of CNS hemorrhage or angiopathy their smaller retrospective cohort (104). Sunitinib carries a NCCN 2B recommendation and a recommendation level C from EANO, though caution is warranted given potential bleeding risk (48, 102).

Meningiomas demonstrate the highest incidence of somatostatin receptor expression of all human tumors, garnering interest in leveraging somatostatin analogues for treatment of refractory lesions (105). The somatostatin receptor subtype overexpressed in 70% of meningiomas, SST_2A_, strongly binds the widely available analog, octreotide (106). *In vitro*, octreotide inhibits meningioma cell proliferation, but does not induce cell death, particularly in cells expressing high levels of the SST_2A_ receptor (107). A prospective pilot study that treated 16 patients with recurrent meningioma with octreotide yielded a radiographic response in 31% of patients, with an additional one-third of patients exhibiting stable disease at 6 months, with minimal associated drug toxicity (108). While this benefit was not observed in a prospective, phase II study of 8 patients with recurrent, treatment-resistant meningioma or hemangiopericytoma, a large retrospective analysis of 43 (only 11 of whom were grade 2 or 3) patients with refractory meningioma treated with octreotide demonstrated improved progression-free survival particularly in skull base lesions (109, 110). Given the possible clinical benefit of octreotide with minimal toxicity, the CNS NCCN guideline classifies it as a level 2B drug for patients with recurrent meningioma.

Disruptions in the mTOR pathway are well documented in high grade meningiomas, with mTOR inhibition associated with decreased proliferation *in vitro* (107, 111). This has resulted in several studies examining combination therapy of everolimus, a small molecule mTOR kinase inhibitor, to other systemic treatments for recurrent meningiomas, including octreotide and bevacizumab (104, 112, 113). The phase II CEVOREM trial of 20 patients reported a median PFS of 6.6 months of the combination of octreotide and everolimus, while a median PFS of 12.1 months was reported by Cardona and colleagues for their retrospective study of 14 patients treated with everolimus, octreotide, and sunitinib (104). Finally, the combination of everolimus and bevacizumab has also been promising, with a median PFS of 22 months in grade 2 and 3 meningiomas (112). Though it remains unclear if there was additional benefit from combinatorial therapy, the combination of everolimus and bevacizumab carries a level 2B recommendation from the NCCN, with multiple mTOR pathway-targeting drugs being actively investigated.

Immunotherapeutic agents have demonstrated mixed efficacy to date in meningioma. Recombinant interferon-α is one such agent that demonstrated *in vitro* inhibition of meningioma cells (114). A prospective study of 35 patients with treatment-refractory WHO grade 1 meningiomas treated with interferon-α had promising results, yielding a median progression-free survival of 7 months. Although no radiographic response was noted in these patients, the study did demonstrate a modest control rate compared to historical controls. Unfortunately, interferon-α had limited efficacy for aggressive, high-grade tumors; a retrospective study of 35 patients receiving interferon-α demonstrated just 17% PFS at 6 months and no evident radiographic response (115). Similarly, immune checkpoint inhibition by the programmed death-1 ligand (PD-L1) pathway (known to be upregulated in high-grade meningiomas) has failed to demonstrate significant response to date, with a recent phase 2 study of nivolumab monotherapy in 25 patients failing to demonstrate improved progression free survival (116). Despite initial setbacks, several immunotherapies remain under active evaluation including nivolumab, pembrolizumab, and avelumab.

Also under investigation for the treatment of meningioma are tyrosine kinase inhibitors. Interest in this category arose from the finding of activated PI3K and MAPK signaling pathways in aggressive meningiomas (117–119). Two such tyrosine kinase inhibitors with proven tolerability and efficacy in other tumors, sorafenib and regorafenib, were shown to impair cell viability and increase apoptosis *in vitro* with meningioma cells and to improve survival in an *in vivo* murine xenograft (120). A 2014 phase II clinical study treated 25 patients with aggressive meningioma with an oral tyrosine kinase inhibitor of VEGFR, vatalanib, with 6 month PFS of 54% (121). There are multiple additional tyrosine kinase inhibitors under investigation, targeting various receptor tyrosine kinase such as EGFR, PDGF, and FGFR with results pending (122–125).

As described above, molecular analysis of meningiomas has identified numerous driver mutations in genes that can be targeted with small molecular inhibitors or other therapeutic strategies. A promising phase 2 clinical trial, NCT02523014, led by Brastianos et al. is currently ongoing with 4 arms (2 closed, 2 ongoing) designed to tailor therapy to the specific molecular alterations identified in patients’ meningiomas (126). Meningiomas with *SMO* or *PTCH1* mutations were treated with vismodegib, an FDA-approved Hedgehog signaling pathway inhibitor. Those with *NF2* mutations received a FAK inhibitor, thought to act as a synthetic lethal with *NF2* loss-of-function, with results from this arm reported as showing improved PFS at 6 months (33%) compared to historical controls with minimal adverse effects (127). Tumors with *AKT1*, *PIK3CA* or *PTEN* mutations are treated with capivasertib, an AKT kinase inhibitor, and those with *CDK4*, *CDK6*, *CDKN2A*, *CCND1*, *CCND2*, *CCND3*, and *CCNE1* treated with abemaciclib, a CDK inhibitor. As additional molecular drivers of meningioma pathophysiology are identified, additional targeted therapies will undoubtedly be revealed for recurrent lesions.

## Conclusions

Although often considered a benign entity, many intracranial meningiomas are anything but, requiring potentially morbid surgical resections and radiation treatments with few viable systemic therapy alternatives. WHO grading predicts aggressiveness of meningiomas relatively well, but as demonstrated in the descriptive case examples, is not perfect, particularly in the broad classes of grade 2 lesions. Recent progress in characterization of the genetic and epigenetic landscape of these lesions may significantly improve our ability to better delineate aggressive tumors. Such tumors may be well-served with immediate postoperative adjuvant therapy or closer monitoring. Finally, improved molecular understanding has permitted targeted therapies including antiangiogenic agents, tyrosine kinase inhibitors, somatostatin inhibitors, and genetically targeted small molecular inhibitors with highly anticipated results from ongoing clinical trials.

## Author Contributions

BP, RD, SP, OB, JH, and AK prepared the manuscript and figures. AK oversaw the project. All authors contributed to the article and approved the submitted version.

## Conflict of Interest

AK is a consultant for Monteris Medical and has received research grants from Monteris Medical for a mouse laser therapy study as well as from Stryker and Collagen Matrix for clinical outcomes studies about a dural substitute, which have no direct relation to this study.

The remaining authors declare that the research was conducted in the absence of any commercial or financial relationships that could be construed as a potential conflict of interest.

## Publisher’s Note

All claims expressed in this article are solely those of the authors and do not necessarily represent those of their affiliated organizations, or those of the publisher, the editors and the reviewers. Any product that may be evaluated in this article, or claim that may be made by its manufacturer, is not guaranteed or endorsed by the publisher.
