# Corrigendum: Identification and management of aggressive meningiomas

**DOI:** 10.3389/fonc.2022.1107271

**Published:** 2022-12-13

**Authors:** Bhuvic Patel, Rupen Desai, Sangami Pugazenthi, Omar H. Butt, Jiayi Huang, Albert H. Kim

**Affiliations:** ^1^Department of Neurological Surgery, Washington University School of Medicine, St. Louis, MO, United States; ^2^Department of Medicine, Division of Medical Oncology, Washington University School of Medicine, St. Louis, MO, United States; ^3^The Brain Tumor Center, Siteman Cancer Center, Washington University School of Medicine, St. Louis, MO, United States; ^4^Department of Radiation Oncology, Washington University School of Medicine, St. Louis, MO, United States

**Keywords:** meningioma, CNS tumors, chemotherapy, radiation therapy, immunotherapy, skull base surgery

## Error in Figure

In the published article, there was an error in **Figure 1**. All locations in the figure that mention “TRAF4” are incorrect and should be changed to “TRAF7”. The corrected **Figure 1** and its caption (unchanged) appear below.

**Figure 1:**
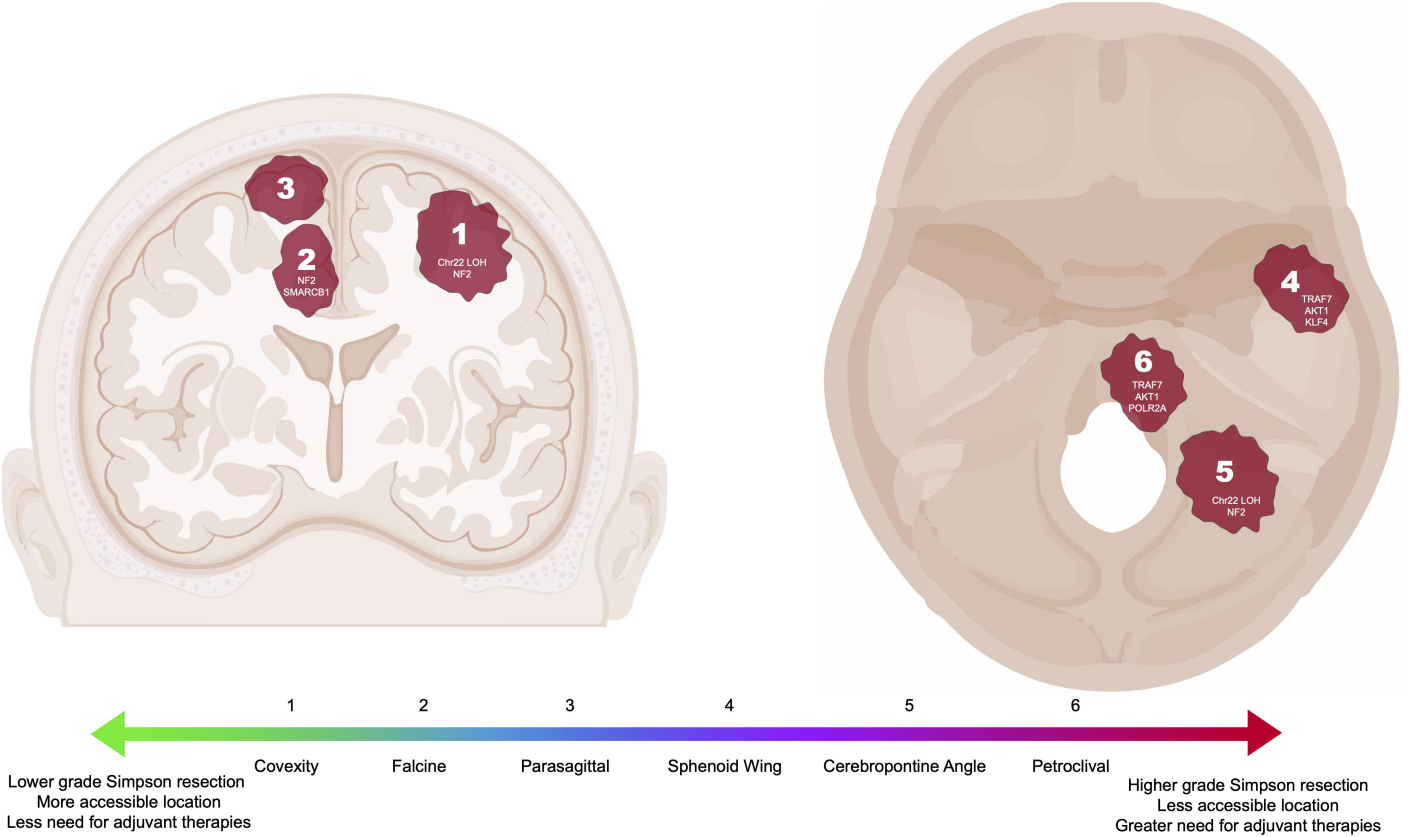
Common intracranial locations of meningiomas highlighted in this review with associated DNA driver mutations or chromosomal loss (6, 14, 23, 24). Locations correlated to a generalized scale ranging from less (green) to more (red) complicated to resect and manage. Meningioma locations not pictured include clinoid, foramen magnum, cavernous sinus, suprasellar, and tentorial.

The authors apologize for this error and state that this does not change the scientific conclusions of the article in any way. The original article has been updated.

## Publisher’s note

All claims expressed in this article are solely those of the authors and do not necessarily represent those of their affiliated organizations, or those of the publisher, the editors and the reviewers. Any product that may be evaluated in this article, or claim that may be made by its manufacturer, is not guaranteed or endorsed by the publisher.

